# Supplementary material for: What does it take to learn the rules of RNA base pairing? A lot less than you may think
Source: bioRxiv. 2025 Aug 2:2025.07.31.668042. Preprint. [Version 1] doi: 10.1101/2025.07.31.668042 (PMC12324431; doi:10.1101/2025.07.31.668042)
Supplement: Supplement 1 [file media-1.gz › supplemental_material/src/rscape_v2.6.0.tar.gz!/rscape_v2.6.0/lib/infernal/Manuscripts/banded-cyk/tbl-emissioncounts.doc]

|  |  | # filtered | # aln | # consensus | # consensus | base pair | SS |
| --- | --- | --- | --- | --- | --- | --- | --- |
| alignment | # seqs | seqs | columns | base pairs | SS columns | counts | counts |
| LSU | 1551 | 139 | 7270 | 601 | 1532 | 65229 | 180558 |
| SSU bap | 12773 | 254 | 2653 | 421 | 680 | 97834 | 153565 |
| SSU euk | 7151 | 207 | 4558 | 407 | 959 | 72521 | 174260 |
| SSU mito | 1039 | 107 | 3791 | 216 | 524 | 19803 | 56510 |

Table 2: **Summary statistics for the dataset used for emission prior estimation.** “SS” = single-stranded.
